# Hydroxysafflor Yellow A Exerts Anti-Inflammatory Effects Mediated by SIRT1 in Lipopolysaccharide-Induced Microglia Activation

**DOI:** 10.3389/fphar.2020.01315

**Published:** 2020-09-11

**Authors:** Xiude Qin, Juanjuan Chen, Guowei Zhang, Chuanpeng Li, Jinqiang Zhu, Hong Xue, Jinfang Li, Tianxiang Guan, Haotao Zheng, Yu Liu, Haobin Cai

**Affiliations:** ^1^Encephalopathy Department, Shenzhen Traditional Chinese Medicine Hospital, The Fourth Clinical Medical College of Guangzhou University of Chinese Medicine, Shenzhen, China; ^2^Department of Traditional Chinese Medicine, College of Traditional Chinese Medicine, Hebei University, Baoding, China; ^3^The 1st Clinical Medical College, Dongzhimen Hospital, Beijing University of Chinese Medicine, Beijing, China; ^4^Institute of Traditional Chinese Medicine, Tianjin University of Traditional Chinese Medicine, Tianjin, China

**Keywords:** hydroxysafflor yellow A, anti-inflammatory, lipopolysaccharide, SIRT1, microglia

## Abstract

Abnormal microglia activation causes sever neuroinflammation, contributing to the development of many diseases, yet the mechanism remains incompletely unknown. In current study, we identified that Hydroxysafflor yellow A (HYA), a chalcone glycoside derived from *Carthamus tinctorius L* effectively attenuates LPS-induced inflammation response in primary microglia *via* regulating the expression of inflammatory genes and remodeling the polarization of microglia. We also reported the effects of HYA on improving lipopolysaccharide (LPS)-stimulated mitochondrial dysfunction and oxidative stress for the first time. Interestingly, we found that HYA could serves as an effective SIRT1 activator. Deficiency of SIRT1 abrogates the protective effects of HYA against LPS-induced response. Overall, our data suggest HYA, a novel SIRT1 activator, could serve as an effective approach to treat LPS-induced neurodegenerative diseases.

## Introduction

Previous studies indicated that abnormal activation of microglia leads to neuroinflammation and contributes to the development of some several diseases, including Parkinson’s disease, Alzheimer’s disease, and other diseases sclerosis and encephalitis (Nolan et al., 2013; Heppner et al., 2015; Borjini et al., 2016; Schwartz and Deczkowska, 2016). Neuroinflammation homeostasis is mainly contributed by the microglia cells, which actively survey the brain micro-environment dependent on its activation. In mature CNS, microglia keeps in apparently dormant state, yet performs its effects to monitoring micro-environment, which benefits for the neurovascular integrity to defend to the potentially damage to the immune systems (Demir et al., 2016). During inflammation exposure, the microglias are over-activated and secrets pro-inflammatory cytokines to neighboring neurons, which is considered as a deleterious risk for the microenvironment health (Hirsch and Hunot, 2009). Thus, several studies indicated that genetic or no-genetic factors which inhibits its activation, have been recognized as main approaches to delay or therapy neuroinflammatory diseases.

SIRT1, which is classed as a nicotinamide adenine dinucleotide (NAD+)-dependent enzyme, belongs to the sirtuin protein family and functions as a crucial enzyme for deacetylating the proteins involved in cellular regulation (Melhem et al., 2016). Accumulating evidence suggested that SIRT1 plays crucial roles in the DNA damage response, carcinogenesis, and regulation of lifespan. Recent studies suggested that AMPK-SIRT1 axis inhibits NF-κB activation and pro-inflammatory cytokines secretion upon its deacetylation activity to protect mice against neuroinflammatory diseases (Parodi et al., 2015). And this process may also attributed by the improvement of redox imbalance, which is dependent on the activation of SIRT1-Nrf2 (Parodi et al., 2015). Moreover, increasing studies demonstrated series chemicals, which acts as SIRT1 modulator, defends against LPS-Induced Inflammatory Responses in mice partly relying on the reduction of reactive oxygen species (ROS), implying the possibility of SIRT1 activator to treat neuroinflammatory diseases (Ullah et al., 2018; Wang S. et al., 2019).

HYA, derived from *Carthamus tinctorius L*, is a chalcone glycoside and previously reported to therapy liver injury dependent on its antioxidant property. More recently, HYA was validated to improve mitochondrial permeability transition pores in rat brain (Tian et al., 2008; Wang et al., 2013; He et al., 2015). Administration with HYA ameliorates myocardial ischemia-reperfusion injury *via* activation of Akt/Nrf2/HO-1 pathway (Hu et al., 2016). In vitro, HYA treatment suppressed oxygen-glucose deprivation induced-inflammatory responses (Li et al., 2013). Previous studies indicated that HYA also exerts anti-inflammatory effects on other tissues and cells, including lung, mesencephalic primary cells, BV2 cells, co-cultured microglia and neurons or macrophages (Zhang et al., 2014; Lv et al., 2016; Wang et al., 2018; Wang et al., 2020). However, whether and how HYA scavenges and attenuates LPS-induced ROS and inflammatory stress remains incompletely unknown.

In present study, we hypothesized that SIRT1 mediates HYA induced protective effects against LPS induced cell injury. An LPS-induced microglial cell models and pharmacological blockade or small interfering RNA (siRNA) of SIRT1 were used to elucidate these hypotheses.

## Methods

### Experimental Materials

LPS (derived from Escherichia coli) obtained from Sigma (St. Louis, MO, United States) at 100 ng/ml for 6 h were used to activate microglia. HYA (purity > 99%) E (HY-N056) at different dosage were used after LPS treatment and Ex-527 (HY-15452) purchased from MCE at 100 nM were used to inhibit SIRT1. Rabbit polyclonal antibodies against SIRT1 (ab220807), Nrf2 (ab62352), SOD (ab80946), Homox 1 (ab133057), and β-actin (ab179467) were purchased from Abcam (Cambridge, United Kingdom). Secondary antibodies of rabbit-goat lgG-horseradish peroxidase (HRP, sc-2054) were purchased from Santa Cruz Biotechnology, Inc (Santa Cruz, CA, United States). Ad-shSIRT1 was purchased from DongBio. Co. Ltd, Shenzhen, China. All the primary antibodies were diluted at 1:1000.

### Cell Culture

Primary rat ventral mesencephalic microglia were isolated and cultured as previously described (Panicker et al., 2015). Briefly, Wistar rats at day 1 to day 2 were used to isolate the cortices of postnatal, containing mixed glial cultures. Cortices were dissociated by 0.25% trypsin followed by trituration. Dissociated cells were seeded into the cell cultured bottles (125 cm^2^) precoated with poly-d-lysine (20 mg/ml). Cells were maintained in DMEM with 10% fetal bovine serum (FBS) and 1% penicillin, streptomycin. After 14-day incubation, the flasks were shaken to separate microglia from astrocytes. The purity of primary microglia was about 98.5%. The BV2 cells were maintained in DMEM medium maintained in DMEM with 10% fetal bovine serum (FBS) and 1% penicillin, streptomycin. The cell viability was measured using a CCK8 assays kit according to the manufacturer’s instructions (CA1210-100 (Solarbio Science&Technology Co, Beijing, China)).

### Ethics Statement

For animal experiments, all animal care and experiments were carried out in strict accordance with the recommendations of the Guide for the Care and Use of Laboratory Animals of the Ministry of Health (China), and approved by the Ethics Committee of Guangzhou University of Chinese Medicine.

### Mitochondrial DNA Copy Number

The mitochondrial DNA (mtDNA) copy number of microglia from indicated groups was used as a marker for mitochondrial density using qPCR (Liu et al., 2015; Cheng et al., 2020). Briefly, total DNA was isolated from microglia cells using a Universal Genomic DNA Extraction kit (Tiangen) according to the manufacturer’s instructions. The ratio of COXII (mitochondrial-encoded gene)/Rps18 was used to show the mitochondrial DNA copy number. The primer sequences of COX II and Rps18 are presented in **Supplemental Table 1**.

### ROS Detection

ROS detection of microglia under different conditions was conducted using ROS assay kit according to the manufacturer’s instructions (KGAF019; KeyGen BioTECH, Jiangsu, China). Briefly, indicated cells were incubated with DCFH-DA with a final concentration of 10 μm for a 0.5-h incubation at 37°C, protected from light. Finally, cells were washed three times with PBS, and then, the emission of fluorescence was detected by fluorescence microscopy and flow cytometry (Shen et al., 2020).

### Molecular Docking

The structure of HYA was obtained from the ChemSpider database of the Royal Society of Chemistry (ChemSpider ID 9797) and drawn using ChemDraw Std 14. The three-dimensional crystal structure of hSIRT1 was obtained from the RCSB Protein Data Bank (PDB code: 3HC5). The cocrystallized structure and docking complex were prepared using SYBYL2.1.1 to correct structural errors, such as broken bonds and missing loops. Docking results were analyzed, and figures were created in Discovery Studio Visualizer.

### RNA Extraction and Quantitative (q) Real-Time PCR

Total RNA was extracted from pulverized cells with TRIzol (Takara), and then reverse-transcribed to cDNA with a high-capacity cDNA reverse transcription kit (Applied Biological Materials Inc, Vancouver, Canada). qPCR (PowerUp™ SYBR™ Green Master Mix) was performed by using gene-specific primers and SYBR Green. mRNA levels for specific genes were normalized by β-actin mRNA levels. The mRNA transcription levels of the target gene in each group of experiments were expressed as a multiple relative to the control groups. All primers are listed in electronic **Supplementary Material Table 1**.

### Western Blot Analysis

Proteins were extracted from cell lysis buffer, and the concentrations of total protein were measured by BSA method. 50 to 80 µg of proteins were loaded into the 10% SDS polyacrylamide gel, and then separated proteins were electrotransferred to polyvinylidene difluoride membranes. Western blot assays were performed using indicated antibodies.

### SIRT1 Deacetylase Activity Assay

Having been divided into 4 groups (in the presence or absence of 10 mM drug HYA, the BV2 cells were, respectively, exposed to PBS, LPS, LPS+M or LPS+H), the total protein of BV2 cells was extracted by using a Protein Extraction Kit (Solarbio, HAOMA BioTECH, Guangzhou, China) as recommended by the manufacturer. Then the deacetylase activity of SIRT1 in total protein of BV2 cells was detected with a SIRT1 activity assay kit (Abcam, ab156065, UK) based on instructions of the manufacturer. Measurements of fluorescence intensity was carried out at 340 nm excitation and 460 nm emission using a microtiter plate fluorometer.

### RNA Interference

shRNA-encoding DNA sequences were synthesized by Invitrogen and constructed into adenovirus plasmids (pAdTrack-U6 vectors), and adenoviruses were generated according to previously described procedures (Luo et al., 2007). A protocol for rapid generation of recombinant adenoviruses using the AdEasy system. Nat Protoc 2:1236–1247). The sequence of small interfering (si)RNA against luciferase was 5′-CTTACGCTGAGTACTTCGA-3′, and the sequence of siRNA against SIRT1 was 5′- TGATTGGCACCGATCCTCG -3’.

### Statistical Analysis

All data are presented as means ± standard error of means (SEM) of more than three independent experiments. Statistical Analysis was performed using PRISM (Version 7.00, GraphPad Software, Inc.). Differences between two groups was performed for statistical significance using the Student’s t test. Differences for multiple comparisons were compared using one-way analysis of variance (ANOVA) (Tukey test or LSD test). (*p < 0.05, **p < 0.01, ***p < 0.001).

## Results

### HYA Supplement Inhibits LPS-Induced NO Generation and Proinflammatory Genes in Primary Microglia

**Figure 1:**
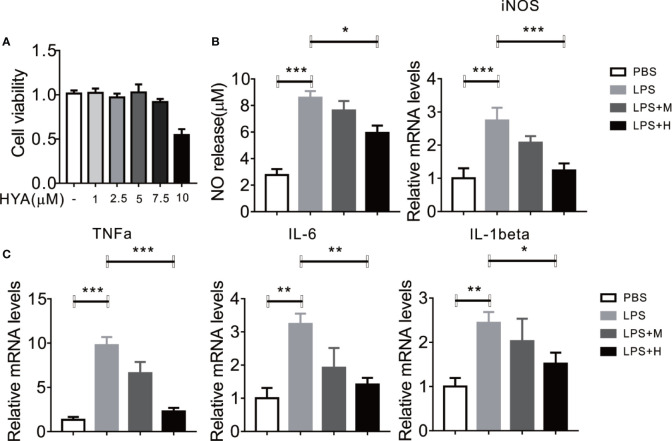
HYA supplement inhibits LPS-induced NO generation and proinflammatory genes in primary microglia. **(A)** Effects of different concentration of HYA on BV2 microglial cell viability. **(B)** Supplement of HYA inhibits LPS-induced NO release and iNOS expression in primary microglial cells. **(C)** The mRNA levels of TNFα, IL-6 and IL-1β were measured by RT-qPCR with different concentration of HYA treatment. M:0.25μM, H:5.0 µM. Student’s *t* test was used to analyze in figure **(A)**. ANOVA was used to analyze in figure **(B**, **C)**. Data are represented as means ± SEM. n = 6 per group. (*P < 0.05, **P < 0.01, ***P < 0.001 compared with the control group).

To determine the direct effects of HYA on anti-neuroinflammation, a different dosage of HYA (0, 1, 2.5, 5, 7.5, 10 µM) were used to test the potential cytotoxicity on primary rat microglial cells. Interestingly, HYA showed a relatively weak cellular toxicity with a comparable safe dosage at 5 µM (**Figure 1A**). Previous studies have shown that activated microglial cells were characterized by the release of various proinflammatory cytokines, NO, and superoxide (Shao et al., 2013). Thus, we test the anti-inflammatory effects of HYA on LPS-activated primary rat microglial cells. Our data indicated that HYA at dosage of 5 µM effectively reduced the LPS-stimulated generation of NO, while a 2.5-µM slight but not significantly affects NO production (**Figure 1B**). We further test the effects of HYA on the inflammatory genes on the LPS treatment. Consistent with our hypothesis, a dose-dependently decreased mRNA expression of iNOS, TNFα, IL-1β, and IL-6 were observed in LPS stimulated primary rat microglial cells (**Figures 1B, C**), implying a strong anti-inflammatory effects of HYA against LPS treatment. As shown in **Figure 1**, a dosage of 5 µM HYA showed most effective inhibition, Therefore, we selected 5 µM in the following experiments.

### HYA Suppressed LPS-Induced M1 Activation and Promoted M2 Polarization in Primary Microglia

**Figure 2:**
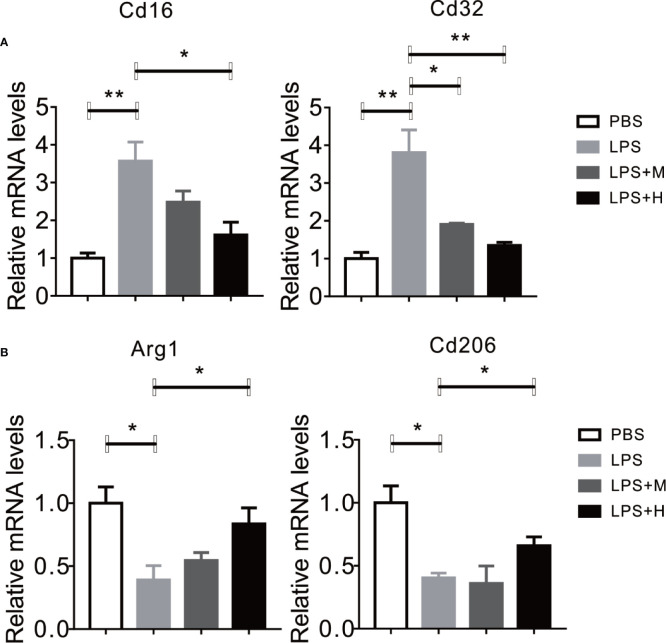
HYA suppressed LPS-induced M1 activation and promoted M2 polarization in primary microglia. **(A)** The expression of M1 markers (CD16 and CD32) were determined by RT-qPCR in different groups. **(B)** HYA treatment significantly promotes the expression of M2 markers (Arg-1 and CD206) in different groups. M: 0.25 µM, H: 5.0 µM. ANOVA was used to analyse. Data are represented as means ± SEM. n= 6 per group. (*P < 0.05, **P < 0.01, compared with the control group).

LPS has been shown to activate microglia cells and drives M0 to M1 dynamic switch, this will lead to the synthesis and release of pro-inflammation cytokines (Yang et al., 2017). Thus, we test whether HYA affects microglia polarization. Consistent with previous studies, LPS treatment stimulated the expression of CD16 and CD32 (M1 markers) in primary microglia, yet HYA supplement significantly rescue these changes, as shown by the down-regulation of M1 markers in a dosage dependent manner (**Figure 2A**). Conversely, LPS treatment markedly inhibits the expression of Arg-1 and CD206 (M2 markers), and HYA treatment effectively reversed LPS induced suppression of M2 markers, suggesting a potential roles of HYA in regulating microglia polarization (**Figure 2B**).

### HYA Treatment Improves LPS-Induced Mitochondrial Dysfunction and Oxidative Stress

**Figure 3:**
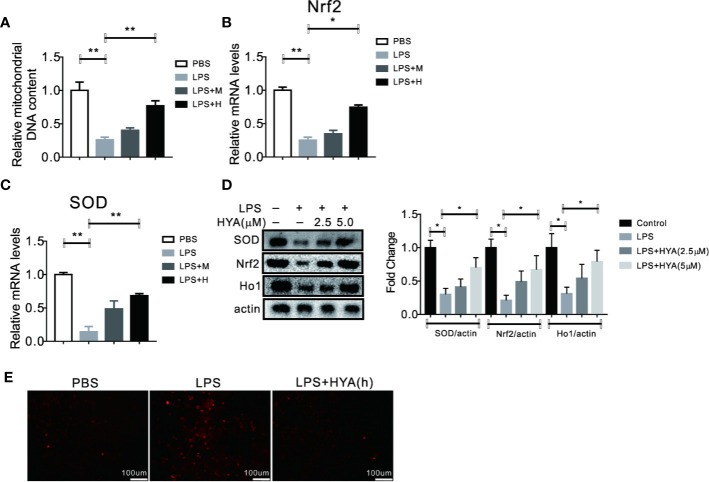
HYA treatment improves LPS induced mitochondrial dysfunction and oxidative stress. **(A)** HYA reversed LPS-stimulated mitochondria loss of primary microglia in a dosage dependent manner. **(B–D)** Genes involved in anti-oxidative stress were measured by RT-qPCR and Western blotting in different groups. **(E)** ROS productions were determined using a ROS assay kit in different groups.M:0.25 µM, H:5.0 µM. ANOVA was used to analyse. Data are represented as means ± SEM. n = 6 per group. (*P < 0.05, **P < 0.01, compared with the control group).

Previous studies indicated that mitochondrial dysfunction and accumulation of excessive peroxides play an important role in LPS induced inflammation of microglia. And mitochondrial transfer effectively ameliorates LPS- induced depression-like behaviors in mice (Wang Y. et al., 2019). Therefore, we hypothesized that HYA treatment could affects LPS-induced mitochondrial dysfunction and ease oxidative stress. To test our hypothesis, mitochondrial numbers and genes involved in anti-oxidative stress, along with ROS production were measured. As expected, our data indicated that HYA significantly rescued the LPS-induced loss of mitochondria of microglia in a dosage dependent manner (**Figure 3A**). Meanwhile, LPS-induced suppression of genes involved in anti-oxidative stress, including Nrf2 and SOD were effectively reversed by HYA both in mRNA and protein levels (**Figures 3B–D**). Of note, HYA (5 µM) markedly decreased the LPS-stimulated ROS generation, suggesting the potential protective effects of HYA on LPS-induced mitochondrial dysfunction and oxidative stress (**Figures 3E** and **S1**).

### HYA Serves as a SIRT1 Activator in Primary Microglia

**Figure 4:**
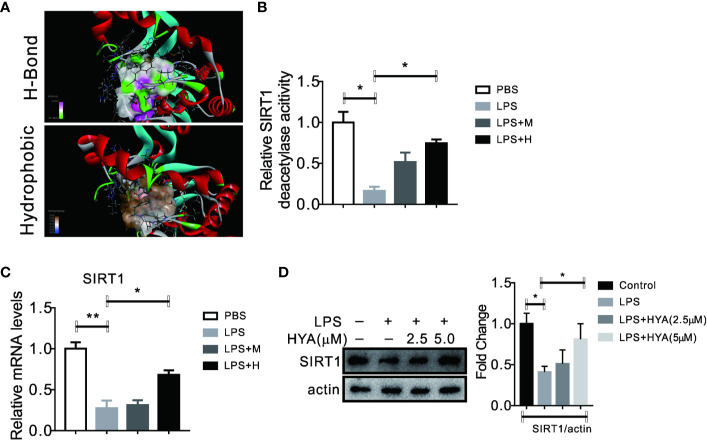
HYA serves as a SIRT1 activator in primary microglia. **(A)** Docking analysis the binding affinity between HYA and SIRT1. **(B)** HYA treatment rescued LPS-stimulated inhibition of SIRT1 enzymes activity. **(C, D)** The effects of HYA on SIRT1 expression both in mRNA and protein levels was measured by RT-qPCR and Western blotting. M: 0.25 µM, H: 5.0 µM. ANOVA was used to analyse. Data are represented as means ± SEM. n = 6 per group. (*P < 0.05, **P < 0.01, compared with the control group).

Accumulating evidences showed that sirtuin proteins manipulates the polarization of microglia, especially SIRT1, a crucial enzyme for deacetylation (Chen et al., 2018). Here, we also test the effects of HYA on SIRT1. Interestingly, our docking analysis between HYA and SIRT1 showed a good binding affinity mainly through stable hydrogen-bond interactions with residues of TYP194, GLN98, SER190, ALA22, ASP32, PHE33, and ASP101 (**Figures 4A** and **S2**). Moreover, this tight binding of HYA could effectively rescued the LPS-induced reduction of SIRT1 deacetylase activity (**Figure 4B**). Additionally, HYA treatment recovers the expression of SIRT1 in primary microglia, even on LPS stimulation state (**Figures 4C, D**), suggesting that HYA could serve as an effective SIRT1 activator.

### Deficiency of SIRT1 Abrogates the Anti-Inflammation Effects of HYA Against LPS Treatment

**Figure 5:**
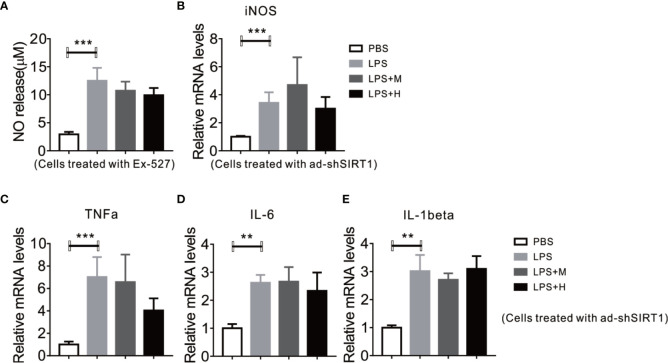
Deficiency of SIRT1 abrogates the anti-inflammation effects of HYA against LPS treatment. **(A)** Ex-527 pretreatment blocks HYA-induced inhibition of NO generation after LPS exposure. **(B, C)** Ad-shSIRT1–mediated deficiency of SIRT1 abrogates HYA-induced suppression of inflammatory genes, including iNOS, TNFα, IL-6, and IL-1β. **(D, E)** The expression of effects M1 markers (CD16 and CD32) and M2 markers (Arg-1 and CD206) were measured by RT-qPCR after ad-SIRT1 infection followed by different concentration of HYA treatment. M: 0.25 µM, H: 5.0 µM. ANOVA was used to analyse. Data are represented as means ± SEM. n= 6 per group. (**P < 0.01, ***P < 0.001 compared with the control group).

**Figure 6:**
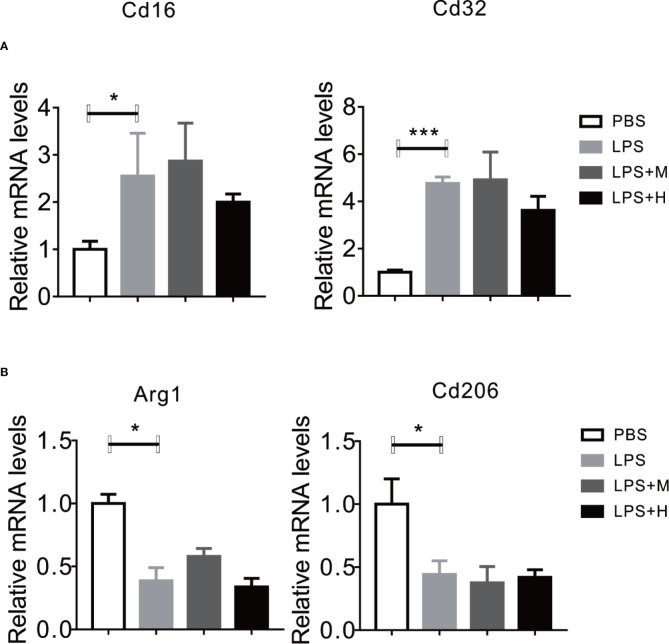
Knockdown of SIRT1 abrogates the effects of HYA on microglia polarization. **(A, B)** The expression of M1 markers (CD16 and CD32) and M2 markers (Arg-1 and CD206) were measured by RT-qPCR after ad-SIRT1 infection followed by different concentration of HYA treatment. M: 0.25 µM, H: 5.0 µM. ANOVA was used to analyze. Data are represented as means ± SEM. n = 6 per group. (*P < 0.05, ***P < 0.001 compared with the control group).

Given the perfect binding affinity between HYA and SIRT1, we hypothesized that SIRT1 may mediates the protective effects of HYA against LPS treatment in primary microglia. As expected, pretreatment with EX-527, an effective SIRT1 blockade, obviously diminished HYA induced recovery of LPS-stimulated NO production (**Figure 5A**). Genes involved in proinflammation cytokines including iNOS, TNFα, IL-1β, and IL-6 were failed to alter by HYA after LPS treatment due to the silence of SIRT1 by ad-shSIRT1 (**Figures 5B–E**), implying a crucial role of SIRT1 in mediating the anti-inflammation effects of HYA against LPS treatment. We further test the requirement of SIRT1 for HYA to regulate microglia polarization. And our data indicated that HYA failed to affect the expression of CD16 and CD32 (M1 markers) and Arg-1 and CD206 (M2 markers) in microglia, even on LPS stimulation status (**Figures 6A, B**).

### Knockdown of SIRT1 Abolished the Recovery of LPS-induced Mitochondrial Dysfunction and Oxidative Stress by HYA

**Figure 7:**
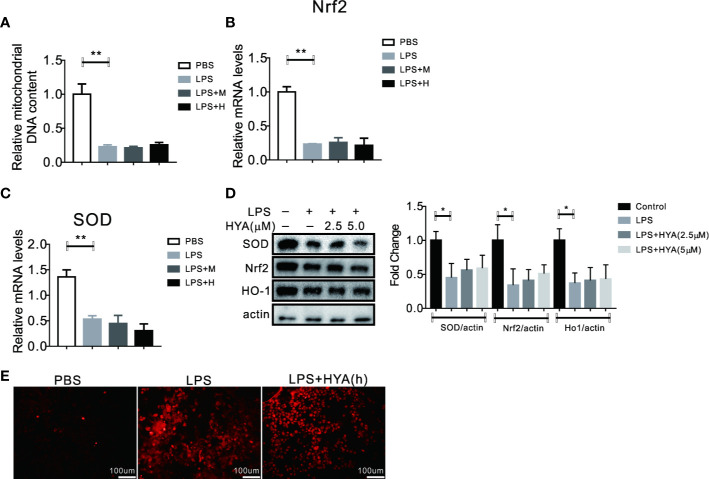
Knockdown of SIRT1 abolished the recovery of LPS-induced mitochondrial dysfunction and oxidative stress by HYA. **(A)** Ad-shSIRT1–mediated knockdown of SIRT1 abolished HYA-induced protection against LPS-stimulated mitochondria loss. **(B–D)** Genes involved in anti-oxidative stress were not altered by HYA due to the deficiency of SIRT1. **(E)** SIRT1 deficiency blocks the protective effects of HYA against LPS-stimulated ROS accumulation, M: 0.25 µM, H: 5.0 µM. ANOVA was used to analyze. Data are represented as means ± SEM. n = 6 per group. (*P < 0.05, **P < 0.01, compared with the control group).

SIRT1 has been previously validate to regulates mitochondria homeostasis and defend LPS induced mitophagy *via* affecting Pink1 and Parkin (Mannam et al., 2014). Here, we also test the role of SIRT1 on HYA induced resistance to LPS-derived mitochondrial dysfunction and oxidative stress. Our data demonstrated that HYA failed to reverse the LPS-induced reduction of mitochondrial number (**Figure 7A**). Meanwhile, LPS-induced inhibition of genes involved in anti-oxidative stress, including Nrf2 and SOD does not alter by HYA both in mRNA and protein levels due to SIRT1 deficiency (**Figures 7B–D**). Interestingly, knockdown of SIRT1 predisposes microglia more sensitive to LPS treatment, as shown by the dramatically increased ROS production. However, HYA treatment failed to rescue the ROS accumulation after LPS exposure, even at a high dosage of 5 µM, suggesting the necessity of SIRT1 for HYA to improve mitochondrial dysfunction and ease oxidative stress in primary microglia (**Figure 7E**).

## Discussion

In the current study, we identified HYA as a novel SIRT1 activator, affecting its transcription levels and enzyme activity. HYA supplement significantly suppressed the LPS-induced activation of microglia and the secretion of pro-inflammatory cytokines. Yet, the anti-inflammatory effects of HYA could be effectively abolished by the SIRT1 antagonist, EX-527, even on LPS-induced state. Further study confirmed that HYA treatment significantly eased the LPS-induced oxidative stress and mitochondrial dysfunction, suggesting a potential mechanism that links anti-oxidative effects to HYA mediated protective effects against LPS-induced cell injury. Of note, the pharmacological blockade and adenovirus-mediated siRNA knockdown of SIRT1 significantly inhibited the protective effects of HYA against LPS-induced excessive generation of ROS, which destroys mitochondrial permeability and structure and aggravates LPS-induced apoptosis and inflammation pressures ultimately. Overall, our findings indicated that HYA could serves as an new SIRT1 activator to defend LPS-induced cell injury in microglia.

Consistent with previous studies, LPS-induced models were used in the present study to activate microglial (Meng et al., 2019). Previous studies have confirmed that LPS, derived from gram-negative bacteria, as its major components of cell wall, could effectively fires cellular inflammatory signaling pathways in microglia cells rather than neighboring neurons and derives inflammatory mediators secretion and release dependent on the toll-like receptors (TLR) (Liu and Bing, 2011; Erny et al., 2015; Woller et al., 2016). This makes LPS suitable to study neuroinflammatory diseases. At same time, multiple studies have confirmed that LPS treatment significantly impaired mitochondria functions in microglia cells and induced ROS over-production, leading to the apoptosis and inflammation eruption (Kim et al., 2015). Evidences have shown that LPS induced oxidative stress plays crucial roles in activating microglia cells (Foresti et al., 2013). In present study, LPS treatment induced microglia over-activation, illustrated by the increased inflammatory genes and proteins, such as TNFα, IL-1β, and iNOS. Meantime, the oxidative burden becomes server, as shown by the accumulation of ROS and reduced mitochondria numbers. Genes involved in the anti-oxidative stress were significantly decreased. These changes were more pronounced in primary cultured microglial cells than BV2 cells, suggesting that cultured microglial cells are more sensitive to LPS stimulation and suitable to use for neuroinflammatory diseases investigation (Henn et al., 2009).

Previous studies indicated that the occurrence of excessive oxidative stress in microglia is closely related to central nervous system injury and infection (O’Leary et al., 2013). Once exposure to the LPS or inflammatory cytokines, microglia was activated in a fast manner, while the mechanism is complex. One acceptable hypothesis is that the LPS treatment could destroy mitochondrial permeability and structure, leading to ROS overproduction and mitophagy (Ashktorab et al., 2004). These will start the inflammation response quickly. When exposure mouse primary microglial cells to cocaine, mitophagy markers were dramatically increased, while the mitochondrial oxygen consumption rate was decreased (Thangaraj et al., 2019). Recent studies confirmed that one reactive oxygen species (ROS)-responsive polymeric micelle system (Ab-PEG-LysB/curcumin (APLB/CUR)) could effectively inhibits microglia over-activation and normalized inflammatory microenvironment and reeducate microglia from an early phase of AD (Lu et al., 2018). SIRT1 belongs to nicotinamide adenine dinucleotide (NAD+)-dependent enzymes and is rich expressed in microglial cells. Evidences confirmed that SIRT1 plays crucial roles in the deacetylating of p65, while SIRT1 deficiency increased the acetylation levels of p65 and NF-κB signaling activity, leading to micoglial M1 activation (Li et al., 2019). Conversely, pharmacological or genetic activation of SIRT1 effectively attenuating inflammation after spinal cord injury and promotes neuronal survival (Chen et al., 2017). Yet, whether and how SIRT1 regulates LPS induced mitophagy remains unknown. In current study, we reported that LPS treatment significantly reduced the expression of SIRT1 and mitochondrial numbers. Consistent with our hypothesis, HYA administration obviously stimulates SIRT1 expression, suggesting a negative effects of HYA on LPS induced cellular mitophagy, mediated by SIRT1.

HYA (HYA) is the major component of *Carthamus tinctorius L* and has previously reported to inhibit adipogenesis *via* stimulating HSL signaling pathways (Zhu et al., 2014). HYA was also confirmed to protect liver from long-term alcohol injury upon enhancing antioxidant capacity and inhibiting the expression of TGF-β (He et al., 2015). Meanwhile, it also defends steroid-induced avascular necrosis of femoral head by suppressing mesenchymal stromal cells activity (Liu et al., 2018) Additionally, previous studies indicated that HYA administration could effectively attenuates Aβ_1-42_ induced inflammation by modulating the JAK2/STAT3/NF-κB pathway (Zhang et al., 2014). Whole brain analysis revealed that HYA treatment attenuates LPS induced neuron damage *via* inhibiting the TLR4 pathway in activated microglial cells (Lv et al., 2016). However, whether and how HYA directly protects microglial cells against LPS treatment remains incompletely unknown. In this study, a direct LPS induced mouse primary microglial cells and BV2 models were used to test the effects of HYA. And we identified HYA as an effective SIRT1 activator both in transcription levels and enzymes levels for the first time. Of note, we found that HYA effectively attenuates mitophagy process in microglial cells to defend LPS-induced inflammation response and oxidative stress in a SIRT1-dependent manner. Overall, our data indicated that HYA could serve as an SIRT1 activator and may be an effective approach to therapy neuroinflammatory diseases in the future.

## Data Availability Statement

The datasets generated for this study are available on request to the corresponding authors.

## Ethics Statement

The animal study was reviewed and approved by Ethics Committee of Shenzhen Traditional Chinese Medicine Hospital, Guangzhou University of Chinese Medicine.

## Author Contributions

XQ conceptualized, planned, and designed the study. JZ, CL, JL, TG, and HX carried out the experiments. XQ and HC drafted and finalized the manuscript. YL assisted in the analysis of data. All authors contributed to the article and approved the submitted version.

## Funding

This study was ﬁnancially sponsored by the Science and Innovation Commission of Shenzhen (JCYJ20180302173504891, JCYJ20190812161807600); the National Natural Science Foundation of China (No. 81904103).

## Conflict of Interest

The authors declare that the research was conducted in the absence of any commercial or financial relationships that could be construed as a potential conflict of interest.
