# Supplementary material for: Hydroxysafflor Yellow A Exerts Anti-Inflammatory Effects Mediated by SIRT1 in Lipopolysaccharide-Induced Microglia Activation
Source: Front Pharmacol. 2020 Sep 11;11:1315. doi: 10.3389/fphar.2020.01315 (PMC7517830; doi:10.3389/fphar.2020.01315)
Supplement: Supplementary file 1 [file DataSheet_1.docx]

Table 1. Primer information for gene amplification

| Primer | Sequences |
| --- | --- |
| TNFα(m) | F1: CAGGCGGTGCCTATGTCTC  R1: CGATCACCCCGAAGTTCAGTAG |
| IL-1β(m) | F1: GAAATGCCACCTTTTGACAGTG  R1: TGGATGCTCTCATCAGGACAG |
| IL-6(m) | F1: CTGCAAGAGACTTCCATCCAG  R1: AGTGGTATAGACAGGTCTGTTGG |
| iNOS(m) | F1: GTTCTCAGCCCAACAATACAAGA  R1: GTGGACGGGTCGATGTCAC |
| β-actin(m) | F1: ATGACCCAAGCCGAGAAGG  R1: CGGCCAAGTCTTAGAGTTGTTG |
| SIRT1(m) | F1: TGATTGGCACCGATCCTCG  R1: CCACAGCGTCATATCATCCAG |
| CD16(m) | F1: AATGCACACTCTGGAAGCCAA  R1: CACTCTGCCTGTCTGCAAAAG |
| CD32(m) | F1: GGAATCCTGCCGTTCCTACTG  R1: ATGGCACAAAGTCCGTGAGAA |
| Arg1(m) | F1: CTCCAAGCCAAAGTCCTTAGAG  R1: GGAGCTGTCATTAGGGACATCA |
| CD206(m) | F1: CTCTGTTCAGCTATTGGACGC  R1: TGGCACTCCCAAACATAATTTGA |
| COX2(m) | F1: ATAACCGAGTCGTTCTGCCAAT  R1: TTTCAGAGCATTGGCCATAGAA |
| Rps18(m) | F1: TGTGTTAGGGGACTGGTGGACA  R1: CATCACCCACTTACCCCCAAAA |





S1.The HYA administration reduced LPS-induced ROS generation detected by flow cytometry. ANOVA was used to analyses. n= 6 per group. Data are means ± SEM. *p < 0.05, **p < 0.01


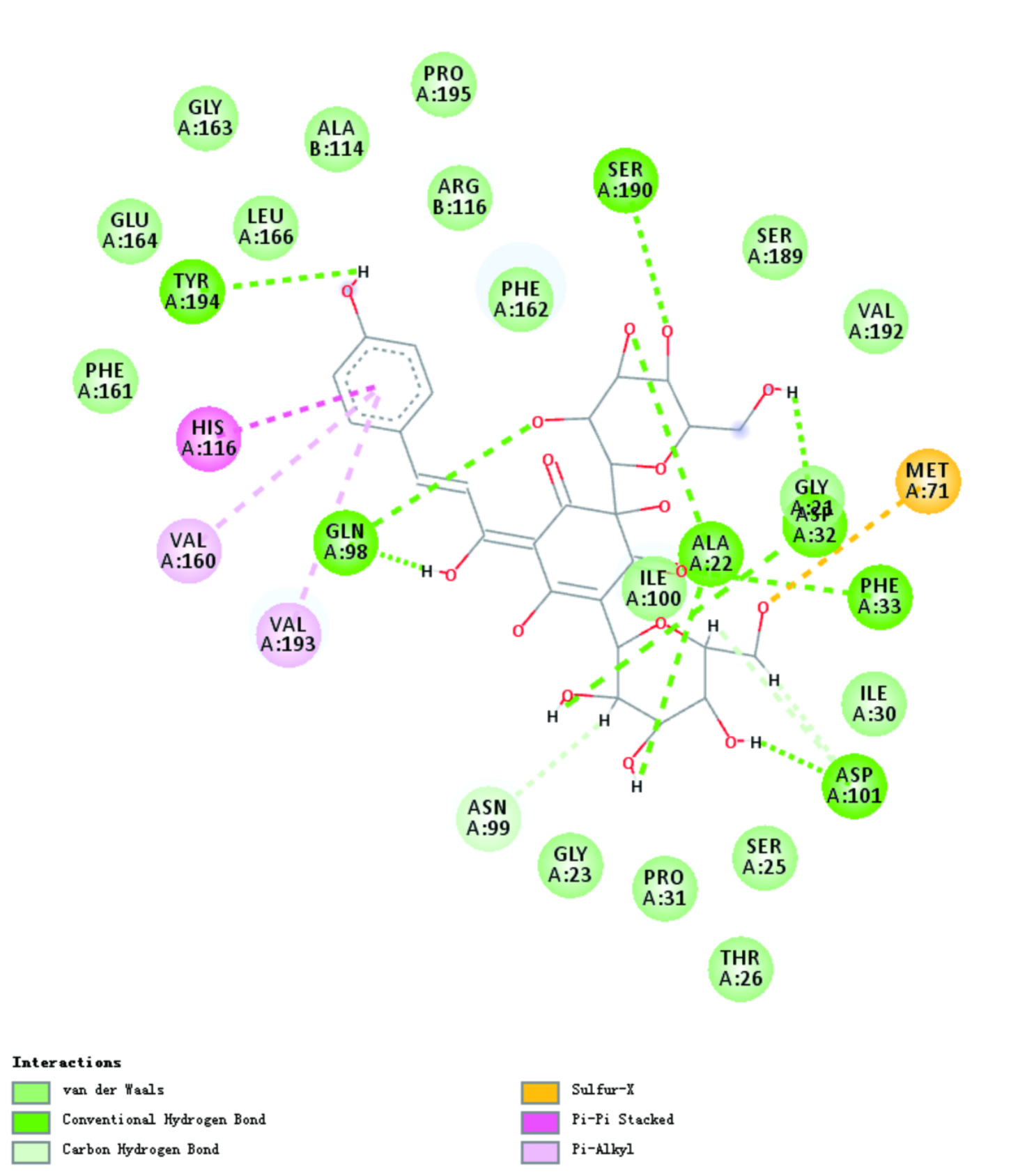


S2.The 2D structure with the predicted binding details of Hydroxysafflor yellow A to hSIRT1 ligand-binding domain (LBD) (rendered in colored sticks).
